# Non-Dipping Blood Pressure or Nocturnal Hypertension: Does One Matter More?

**DOI:** 10.1007/s11906-023-01273-1

**Published:** 2023-11-13

**Authors:** Amber Tang, Eugene Yang, Joseph E. Ebinger

**Affiliations:** 1 Department of Medicine, University of California, Los Angeles, CA USA; 2 Division of Cardiology, University of Washington School of Medicine, Seattle, WA USA; 3 Department of Cardiology, Smidt Heart Institute, Cedars-Sinai Medical Center, Los Angeles, CA USA

**Keywords:** Nocturnal hypertension, Non-dipping, Ambulatory blood pressure monitoring

## Abstract

**Purpose of Review:**

Nocturnal hypertension and non-dipping are both associated with increased cardiovascular risk; however, debate remains over which is a better prognosticator of cardiovascular outcomes. This review explores current literature on nocturnal hypertension and non-dipping to assess their relationship to cardiovascular disease and implications for clinical practice.

**Recent Findings:**

While current data remain inconclusive, some suggest that nocturnal hypertension is a more reliable and clinically significant marker of cardiovascular risk than non-dipping status. Importantly, reducing nocturnal HTN and non-dipping through chronotherapy, specifically evening dosing of antihypertensives, has not been conclusively shown to provide long-term cardiovascular benefits.

**Summary:**

Recent data suggests that non-dipping, compared to nocturnal hypertension, may be falling out of favor as a prognostic indicator for adverse cardiovascular outcomes. However, additional information is needed to understand how aberrant nighttime blood pressure patterns modulate cardiovascular risk to guide clinical management.

## Introduction

The global prevalence of hypertension has doubled in the past several decades with less than a quarter of those with hypertension reaching blood pressure (BP) targets for control [1]. Greater understanding of the pathophysiologic mechanisms linking hypertension with adverse cardiovascular (CV) outcomes has resulted in increased attention to controlling BP throughout the day, rather than relying on isolated clinic measurements. This transition to ambulatory BP assessment—particularly use of ambulatory and home BP monitoring—has driven enhanced recognition of nocturnal hypertension and abnormal nocturnal dipping patterns. Specifically, BP should decrease at night, resulting in a physiologic nocturnal dipping pattern. As such, while contemporary goals aim to achieve a BP of < 130/80 mmHg during the day [2•], nocturnal BP thresholds are much lower. Notably, while most studies define nocturnal hypertension as a nighttime BP ≥ 120/70 mmHg, more aggressive thresholds have been proposed in clinical guidelines, such as > 110/65 mmHg [2•, 3, 4•] . Importantly, prior work suggests that elevated nighttime BP may be a more clinically useful predictor of CV disease than daytime levels, in part due to fluctuations from physical activity and emotional stressors that may reduce reliability of daytime BP measurements [5, 6].

**Fig. 1:**
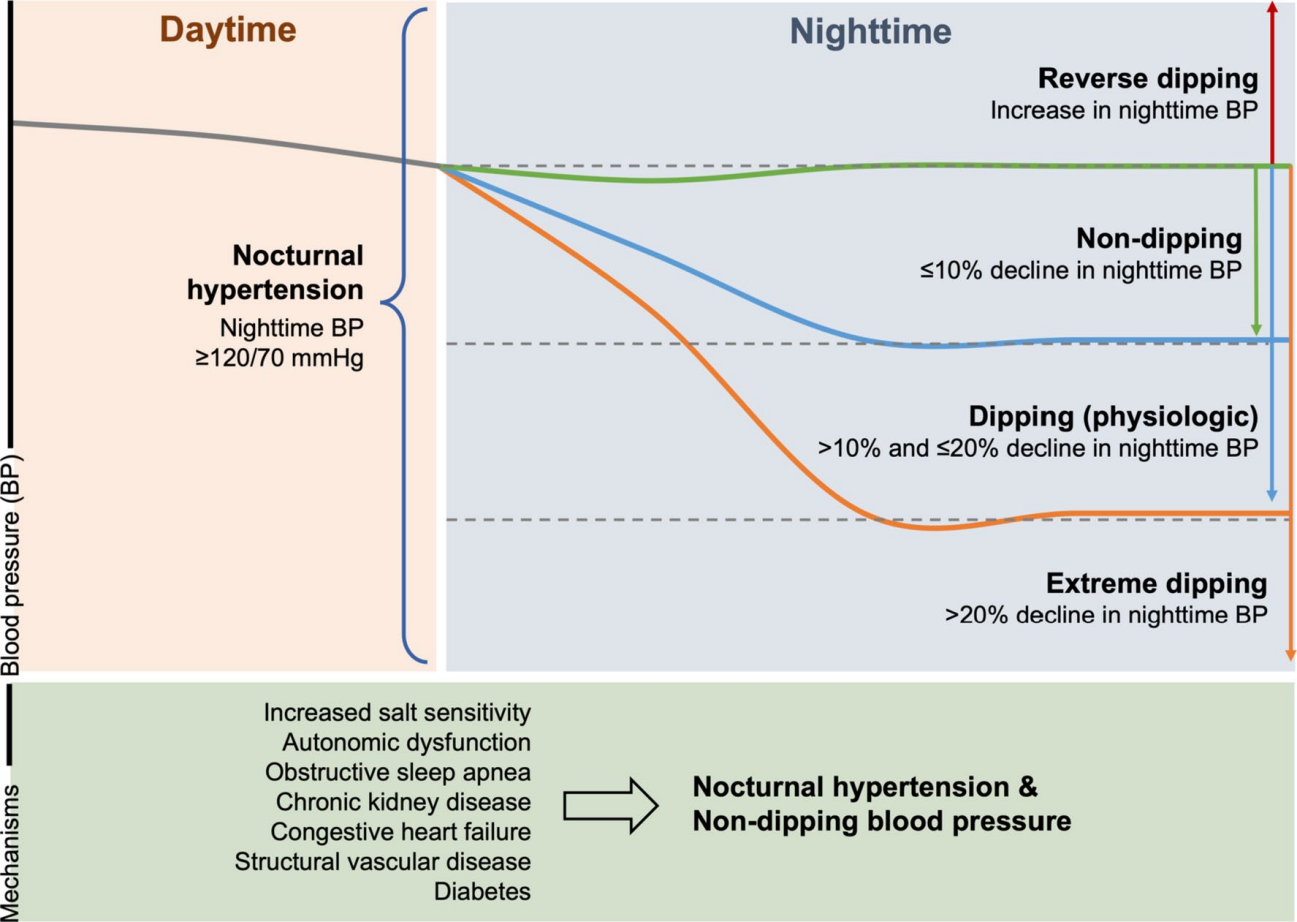
Definitions and potential mechanisms of nocturnal hypertension and different nighttime dipping patterns

Researchers and clinicians have also identified four unique patterns in BP change from day to night: dipping, extreme dipping, non-dipping, and reverse dipping. “Dipping,” first described in the 1980s by O’Brien et al. [7], refers to a decline in nighttime BP of 10–20% compared to daytime levels and is considered physiologic. Arising from this early definition, “extreme dipping” is defined as a > 20% decline, “non-dipping” a < 10% decline, and “reverse dipping” an increase in nighttime BP relative to daytime (Fig. 1) [8]. While some studies have reported that non-dipping patterns are associated with increased risk of adverse CV events, others have suggested that non-dipping is unreliable and a poor prognostic marker of CV risk compared to the use of strict cutoffs for nocturnal hypertension. As a result, there remains ongoing debate regarding whether non-dipping is a clinically significant predictor of CV disease.

## Pathophysiology

The physiologic pathways underlying nocturnal hypertension and non-dipping are not well understood. Several potential mechanisms have been proposed. High salt intake, particularly among salt-sensitive individuals, contributes to both nocturnal hypertension and non-dipping patterns due to preferential excretion of excess sodium at night. Prior studies have found that nocturnal hypertension is associated with low daytime and high nighttime urinary sodium excretion [9, 10]. Dietary sodium restriction was able to restore normal nocturnal dipping patterns among patients who were sodium-sensitive [11]. Non-dipping has been attributed to dysregulation of the autonomic nervous system, including a decreased nighttime parasympathetic response [12, 13]. Compared to dippers, non-dippers were found to have a blunted reduction in urinary catecholamine excretion from day to night as well as greater α1 adrenergic receptor sensitivity, which may be associated with increased nighttime vasoconstriction [14].

Aging and sleep quality, as well as comorbidities including diabetes, heart failure, obesity, and chronic kidney disease, may affect nocturnal BP [15]. Obstructive sleep apnea (OSA) has been extensively linked to nocturnal hypertension and non-dipping as intermittent hypoxia and sleep fragmentation result in sympathetic excitation and subsequent vasoconstriction. Upregulation of the renin-angiotensin-aldosterone system, as well as increased oxidative stress and endothelial dysfunction, in OSA may also contribute to nocturnal hypertension and non-dipping [16–18].

## Nocturnal Hypertension

Nocturnal hypertension is highly prevalent, with studies suggesting that it impacts 40–60% of the population [19, 20]. Moreover, significant disparities have been described, with Black people experiencing a nearly two-fold greater prevalence of nocturnal hypertension compared to white people [21•]. Nearly three-fourths of Black people with diabetes in the Jackson Heart Study were found to have nocturnal hypertension, suggesting even higher rates among specific at-risk populations [22].

The association between nocturnal hypertension and end organ damage is well described. In a large prospective study, patients with sustained hypertension during both day and night were found to have significantly higher urine albumin-to-creatinine ratios compared to participants with isolated daytime hypertension [23•]. A 2016 meta-analysis found that left ventricular (LV) mass and carotid intima-media thickness were significantly greater in patients with nocturnal hypertension compared to nocturnal normotension [24]. Isolated nocturnal hypertension has been associated with significantly higher risk of adverse CV events (HR 1.38, 95% CI 1.02–1.87) and overall mortality (HR 1.29, 95% CI 1.01–1.65) at 7.6 year follow-up, even after adjustment for age and other comorbidities. Day to night sustained hypertension was also associated with increased risk of CV events as well as overall and CV mortality [25]. Notably, there was no significant difference in CV events between normotensive patients and those with isolated daytime hypertension [23•].

## Non-Dipping Blood Pressure

Non-dipping BP describes people who have an attenuated physiologic decrease in nighttime BP (< 10% decline compared to daytime). In a cohort from the Spanish Society of Hypertension Ambulatory Blood Pressure Monitoring Registry, half of untreated patients with nocturnal hypertension were characterized as dippers and one-third as non-dippers. In contrast, there was a higher prevalence of non-dippers (39.4%) among patients receiving antihypertensive therapies, which was attributed to a diminished day-to-night BP ratio with traditional morning antihypertensive dosing [8]. Among both treated and untreated patients, non-dippers were typically older and more likely to be female. They were also more likely to be obese and have a longer duration of hypertension compared to dippers. Comorbidities including OSA, diabetes, CV disease, and renal disease were associated with non-dipping [8, 26].

Similar to nocturnal hypertension, non-dipping status is also associated with significant racial disparities. The Jackson Heart Study, which included Black patients with hypertension, found that nearly two-thirds of participants were non-dippers regardless of antihypertensive medication use. Psychosocial factors, such as depressive symptoms and low perceived social support, were associated with attenuated nighttime BP dipper response [27]. Results from the CARDIA study also found that non-Hispanic Black individuals had a 1.5 times greater prevalence of non-dipping compared to white men and women [21•].

Numerous studies have found that non-dipping is associated with elevated long-term CV risk. In a multicenter, retrospective cohort study, non-dipping was a significant predictor of CV outcomes at a median follow-up of 5.7 years [28]. Several other studies found similar results at 6-year and 9-year follow-up (HR 2.22, 95% CI 1.64–2.95 and OR 2.27, 95% CI 1.41–3.66, respectively). While there was attenuation in adverse CV outcomes after adjusting for comorbid conditions, these associations remained significant (HR 1.96, 95% CI 1.43–2.84 and OR 1.50, 95% CI 1.15–4.30 respectively) [24, 26, 29]. Non-dipping has also been linked to impaired LV mechanics. In a recent study, LV global longitudinal and circumferential strain, as well as endocardial and epicardial strain were significantly reduced among non-dippers and reverse dippers [30]. Furthermore, the CARDIA study found that attenuated nocturnal dipping among young adults was associated with impaired executive function later in life as measured by Stroop testing, a measure of frontal lobe function using complex visual stimuli. Notably, other tests of executive function, such as measures of attention and working memory, were not significantly associated with nocturnal BP [31].

## Reverse Dippers

Reverse dipping is defined as an increase in nighttime BP relative to daytime BP. Some studies suggest a prevalence of around 6% of untreated individuals with nocturnal hypertension. In contrast, 13.5% of those receiving antihypertensive therapies were found to be reverse dippers, which was attributed to administration of antihypertensive medications in the morning, resulting in a decrease in day to night BP ratio [8].

It has been suggested that reverse dipping is a stronger predictor of CV risk than non-dipping. In one study, although both non-dipping and reverse dipping were significantly associated with CV events, reverse dippers had a higher incidence of adverse CV events after a median 5.7-year follow-up compared to non-dippers (17.6% vs 9.5%, respectively) [28]. In contrast, a recent meta-analysis found that among untreated individuals with hypertension, reverse dipping was associated with increased CV event risk, including stroke, while no significant association was observed among non-dippers [32]. In hypertensive patients without baseline CV disease, non-dippers and dippers had similar all-cause mortality and CV events (including sudden death and myocardial infarction) after controlling for covariates. However, reverse dippers had significantly higher rates of CV events at 6.5-year follow-up compared to dippers (HR 1.83, 95% CI 1.26–2.65, *p* < 0.001) [33] Similarly, while both non-dipping and reverse dipping were associated with abnormal LV mechanics, such as reduced LV longitudinal strain, only reverse dipping was associated with reduced circumferential strain and reduced right ventricular global longitudinal strain [30, 34].

Given conflicting findings across studies and methodologic differences, the Ambulatory Blood Pressure Collaboration in Patients with Hypertension conducted a large meta-analysis, which included over 17,000 patients with hypertension. After adjusting for 24-h systolic BP, reverse dipping was significantly associated with adverse CV events (HR 1.79, 95% CI 1.43–2.22), CV mortality (HR 1.84, 95% CI 1.08–3.15), and all-cause mortality (HR 1.73, 95% CI 1.01–2.95). While non-dipping was associated with CV outcomes (HR 1.27, 95% CI 1.06–1.53), there was no difference in mortality compared to dipping [35••].

## Nocturnal Hypertension Versus Non-Dipping Blood Pressure

Despite studies suggesting that both nocturnal hypertension and non-dipping are predictors of future CV events, some have questioned the clinical and prognostic utility of non-dipping. Criticisms of non-dipping include poor reproducibility due to its dependence on daytime BPs, which are subject to significant fluctuations. For example, nocturnal dipping patterns were found to be inconsistent and poorly reproducible over a 5-month period in a study of more than 200 patients followed with 24-h ambulatory BP monitoring. Nocturnal systolic BP decline had a poor intraclass correlation coefficient of 0.48 across measurements, and about a third of patients converted to a different dipping pattern from their initial classification across the study duration [36•]. Another study reported that different definitions of awake and sleep states also led to significant variability in the prevalence of dipping and non-dipping [37]. Furthermore, there is often significant overlap between nocturnal hypertension and non-dipping, making it difficult to isolate the downstream effects of one versus the other. Additionally, analysis of data from the CARDIA Study found an association between the prevalence of LV hypertrophy and elevated BP (both daytime and nighttime) but not with nocturnal dipping status; these results indicate that adverse cardiac remodeling may be driven more by BP level than the percent reduction achieved in BP while asleep [38].

As a result, several studies have suggested that using strict cutoffs for nocturnal hypertension is more significantly associated with CV risk than non-dipping. As shown in Table 1, most studies use a cutoff of ≥ 120/70 mmHg to define nocturnal hypertension. In one cross-sectional study, nocturnal hypertension was significantly associated with LV hypertrophy (OR 11.1, 95% CI 3.0–40.1), while non-dipping was not [39]. Among untreated patients with nocturnal hypertension, both dippers and non-dippers had a similar prevalence of LV hypertrophy and carotid atherosclerosis [40]. Another study found that nocturnal hypertension was more strongly associated with carotid intima-media thickness and LV mass index compared to non-dippers [41, 42].

**Table 1:** Studies comparing cardiovascular risk associated with nocturnal hypertension and non-dipping

**Study**	***N***	**Nocturnal HTN definition**	**Non-dipping definition**	**Primary outcome(s)**	**Primary outcome findings**
Perez-Lloret et al. [39]	223 Cross-sectional	Mean nighttime BP ≥ 120/70	Nocturnal SBP fall < 10% of mean daytime SBP	LVH	**Nocturnal HTN:** OR 11.1 [3.0–40.1] **Non-dipping**: OR 1.4 [0.4–5.5]
Androulakis et al. [41]	319 Cross-sectional	SBP > 120 and/or DBP > 70	< 10% nocturnal decrease in SBP	Vascular indices of organ damage, cardiac structural and functional indices^b^	**Nocturnal HTN vs normotension:** IMT (774 vs 693, *p* = 0.016) LVMI (88.1 vs 82.8, *p* = 0.043) LAD (44.2 vs 39.5, *p* = 0.065) **Non-dipping vs dipping:** IMT (764 vs 758, *p* = 0.889) LVMI (89.6 vs 85.3, *p* = 0.078) LAD (36.4 vs 36.6, *p* = 0.895)
Koroboki et al. [42]	937 Cross-sectional	SBP ≥ 120	Mean reduction in nighttime SBP < 10% compared to daytime	LVM	**Nocturnal HTN:** independent determinant of LVM (*p* = 0.037) **Non-dipping**: not an independent determinant of LVM (*p* = 0.136)
de la Sierra et al. [43]	99,884 Cross-sectional	SBP ≥ 120	≤ 10% reduction in nighttime SBP compared to daytime	Cardiovascular risk factors (DM, creatinine, microalbuminuria, LVH)^a^	**Group 1: nocturnal normotension + dipping** DM % (10.0), creatinine (79.6 ± 28.3), microalbuminuria % (7.6), LVH % (1.2) **Group 2: nocturnal normotension + non-dipping** DM % (12.4)^b^, creatinine (82.3 ± 22.1), microalbuminuria % (9.3)^b^, LVH % (1.9)^b^ **Group 3: nocturnal hypertension + dipping** DM % (13.9)^b^, creatinine (80.3 ± 22.1), microalbuminuria % (11.3)^b, c^, LVH % (2.1)^b^ **Group 4: nocturnal hypertension + non-dipping** DM % (16.8)^b, c, d^, creatinine (80.4 ± 27.4)^b^, microalbuminuria % (14.6)^b, c, d^, LVH % (2.6)^b^

All studies found directly comparing nocturnal hypertension and non-dipping blood pressure were greater than 5 years old

*BP* blood pressure, *DBP* diastolic blood pressure, *DM* diabetes mellitus, *HTN* hypertension, *IMT* intimamedia thickness, *LAD* long axis left atrial diameter, *LVH* left ventricular hypertrophy, *LVM* left ventricular mass, *LVMI* left ventricular mass index, *OR* odds ratio, *SBP* systolic blood pressure

^a^Additional variables in full article

^b﻿^*p* < 0.05 compared to group 1

^c^*p* < 0.05 compared to group 2

^d^*p* < 0.05 compared to group 3

Other studies have suggested that non-dipping may be an indicator of more advanced disease among patients with nocturnal hypertension. In a study on CV risk profiles among nocturnal hypertension and non-dipping patients, those with concurrent nocturnal hypertension and non-dipping had higher levels of microalbuminuria and reduced renal function compared to patients with nocturnal hypertension without concomitant non-dipping or non-dipping without nocturnal hypertension [43]. Additionally, in a cohort of untreated individuals with hypertension, dippers with normal nocturnal BPs had a lower 10-year CV risk score compared to both non-dippers and dippers with nocturnal hypertension (*p* = 0.050) [44].

## Therapeutic Implications

Given the elevated CV risk associated with nocturnal hypertension, many have suggested that bedtime dosing of antihypertensive medications may improve nighttime BP control. In a study of patients randomized to either daytime or nighttime administration of valsartan 160 mg for 3 months, nighttime dosing resulted in a significant increase in the diurnal to nocturnal BP ratio compared to those treated upon awakening. Nighttime administration significantly reduced the number of patients classified as non-dipping (73.1% vs 12.5%, *p* < 0.001). Notably, there was no significant difference in the rate of extreme dipping in either treatment group [45]. Similar findings were reported with administration of telmisartan 80 mg, which was found to decrease the prevalence of non-dipping from 34 to 8% among those in the bedtime dosing group (*p* < 0.001) [46]. Combination calcium-channel blocker/angiotensin receptor blocker therapies were similarly found to reduce nocturnal BP and increase rates of physiologic dipping patterns when administered at night [47]. One study reported a reduced urinary albumin-to-creatinine ratio among those randomized to bedtime dosing of amlodipine/olmesartan therapy [48]. In a cohort of resistant hypertension patients, all participants had one of their three antihypertensive medications randomized to either nighttime or morning administration. Those in the nighttime dosing arm had significantly lower ambulatory BPs as well as significantly higher day to night ratios of systolic BP (*p* < 0.001) [49]. A recent meta-analysis found that bedtime antihypertensive medication administration resulted in a 4.7-mmHg decline in nocturnal systolic BP and 3.6-mmHg decline in nocturnal diastolic BP, without a significant change to diurnal BP [50].

Despite studies suggesting nighttime administration of BP medications reduces nocturnal BP and produces more of a physiologic dipping pattern, there has been controversy regarding whether chronotherapy (treatment aimed at restoring normal circadian patterns) improves long-term CV risk. Two frequently cited studies evaluating the effects of antihypertensive timing on CV events are the MAPEC and Hygia trials (Table 2). The MAPEC study randomized over 2000 individuals to take all their prescribed antihypertensives either on awakening or at least one at bedtime. At a median follow-up of 5.6 years, the bedtime group had a significantly lower risk of adverse CV events (HR 0.39, 95% CI 0.29–0.51) [51]. The Hygia trial had a similar design and included over 19,000 participants with hypertension. At long-term follow-up, those randomized to the bedtime treatment regimen also had a significantly lower risk of adverse CV events (HR 0.55, 95% CI 0.50–0.61) [52].

**Table 2:** Randomized controlled trials on management of nocturnal hypertension and long-term cardiovascular outcomes

**Study**	***N***	**Cohort**	**Intervention**	**Median follow-up**	**Mean nighttime SBP at follow-up (mmHg)**	**Non-dippers at follow-up (%)**	**Primary outcome**	**Primary outcome findings**
MAPEC [51]	2201	Untreated or resistant HTN	Monotherapy in the morning or bedtime (untreated); alteration of one antihypertensive and randomization of ≥ 1 medication to bedtime (resistant HTN)	5.6 years	116.1 (morning) vs. 110.9 (night), *p* < 0.001	61.6 (morning) vs. 34.4 (night); *p* < 0.001	CV events or all-cause mortality	HR 0.39 [0.29–0.51], *p* < 0.001
Hygia [52]	19,084	Untreated or treated HTN	Ingestion of all antihypertensives in the morning or ≥ 1 antihypertensive at bedtime	6.3 years	118.0 (morning) vs. 114.7 (night), *p* < 0.001	50.3 (morning) vs. 37.5 (night); *p* < 0.001	CV events or cardiovascular-related mortality	HR 0.55 [0.50–0.61], *p* < 0.001
TIME [57]	24,610	HTN on ≥ 1 antihypertensive)	Ingestion of all antihypertensives in the morning or all in the evening	5.2 years	132 (morning) vs. 133 (evening), *p* < 0.0001^a^		Vascular death or hospitalization for non-fatal MI or non-fatal stroke	HR 0.95 [0.83–1.10], *p* = 0.53
BedMed^b^ [58]	Ongoing	HTN on ≥ 1 antihypertensive	Ingestion of all antihypertensives in the morning or all in the evening				Composite all-cause death or hospitalization for MI, ACS, stroke, or CHF	

Design of all listed trials was a prospective randomized open-label, blinded end-point study

*ACS* acute coronary syndrome, *CHF* congestive heart failure, *CV* cardiovascular, *HTN* hypertension, *HR* hazard ratio, *MI* myocardial infarction, *SBP* systolic blood pressure

^a^Self-reported via home blood pressure monitors vs. ambulatory blood pressure monitors used in other studies

^b^Final results not yet published

However, both the MAPEC and Hygia studies have been criticized due to methodologic concerns, including ambiguous descriptions of randomization protocols and unclear reporting of follow-up and adherence [53]. The MAPEC trial also found a greater than 60% relative reduction in CV events among the nighttime-dosing arm, despite a small difference in mean systolic BP between groups at follow-up (122.1 vs 120.8 mmHg in the morning and bedtime arms respectively). These findings are disproportionate to many other outcome studies based on change in BP and reduction in CV events [54]. Similarly, the Hygia study showed a 50% reduction in CV events in the bedtime administration group, with only a minimal difference between study arms in the 48-h ambulatory BP change at follow-up (125.6 vs 124.3 mmHg in the morning and bedtime arms respectively) [55••, 56].

To address the controversies surrounding the MAPEC and Hygia studies, the recently published UK-based TIME trial included over 20,000 adults with hypertension and randomized them to either morning or evening administration of their usual antihypertensive medications. At a median follow-up of 5 years, there was no significant difference in major CV outcomes, including CV-related mortality or hospitalization for myocardial infarction or stroke (3.4% in the morning group vs 3.7% in the evening group, *p* = 0.53) [57••]. The BedMed trial is a similar ongoing Canadian-based study that will assess timing of antihypertensive medication administration on all-cause mortality and CV outcomes [58]. A recently released consensus document from the European Society of Hypertension, International Society of Hypertension, and World Hypertension League stated that given methodologic limitations of the aforementioned studies, current evidence is inconclusive on whether bedtime administration of antihypertensives significantly impacts CV outcomes. They further recommended against routine use of nighttime antihypertensive dosing until additional data becomes available [55].

Given the close link between OSA and nocturnal hypertension, treatment of OSA with continuous positive airway pressure (CPAP) has been suggested to improve both nocturnal and diurnal BP control [18, 59]. CPAP use was shown to markedly reduce nocturnal sympathetic activation as measured by sympathetic nerve activity through multiunit recordings of a muscle nerve fascicle. However, a significant decline was only seen with sustained CPAP use for at least 6 months [60]. In a large meta-analysis, CPAP use resulted in a statistically significant 3.8-mmHg reduction in nighttime systolic BP. A greater decline in overall BP was found among those with more severe OSA at baseline [61].

The SACRA Study also found that empagliflozin 10 mg significantly reduced 24-h systolic BP compared to placebo (− 7.7 mmHg, *p* = 0.002) among patients with type 2 diabetes and uncontrolled nocturnal hypertension, but did not significantly reduce nighttime systolic BP (− 4.3 mmHg, *p* = 0.159) [62]. Of note, oral melatonin has also been found to reduce nocturnal BP with one meta-analysis showing a nearly 5-mmHg drop in systolic BP. However, evidence is scarce, and existing randomized controlled trials examining the benefits of melatonin on nocturnal hypertension have small sample sizes [63].

## Conclusions

Nocturnal hypertension and non-dipping are highly prevalent and associated with increased CV risk. However, given inconsistencies across studies, the clinical significance and management of these abnormal BP patterns remain uncertain. Some investigators have suggested that non-dipping is an unreliable prognostic indicator and may be inferior to nocturnal hypertension alone, as defined by a nighttime BP ≥ 120/70 mmHg. However, relatively few studies directly compare nocturnal hypertension and non-dipping. Reverse dipping has also emerged as a potential clinical predictor of CV events, with some studies even suggesting that reverse dipping is more significantly associated with adverse CV outcomes compared to non-dipping. Chronotherapy has recently received more attention as a means of improving control of nocturnal hypertension and non-dipping without significantly impacting overall 24-h BP control. However, controversial findings from recent studies have raised questions about the long-term benefits of bedtime dosing of antihypertensive therapies. The jury is still out regarding the prognostic value of nocturnal hypertension or non-dipper status on CV events. More prospective, randomized trials are needed to fully understand the clinical significance and tailored management of nocturnal hypertension and non-dipping BP patterns.
